# Interpretable machine learning prioritizes transcriptomic features associated with ferroptosis-mitochondrial permeability transition net activity states in cervical cancer

**DOI:** 10.3389/fgene.2026.1930345

**Published:** 2026-10-05

**Authors:** Huiying Zhang, Yidong Zhu, Weijie Shao, Kai Xu

**Affiliations:** 1 Department of Pain Medicine, Shaoxing People’s Hospital, Shaoxing, China; 2 Hangzhou Sun Taihe Traditional Chinese Medicine and Pharmacy, Hangzhou, China; 3 Hangzhou Fuyang Hospital of TCM Orthopedics and Traumatology, Hangzhou, China; 4 Reproductive Center, The Second Affiliated Hospital of Zhejiang Chinese Medical University, Hangzhou, China

**Keywords:** cervical cancer, ferroptosis, HPV, machine learning, mitochondrial permeability transition, pathway scoring, shap, XGBoost

## Abstract

**Background:**

High-risk human papillomavirus (HPV)-associated cervical carcinogenesis involves oxidative stress, mitochondrial dysfunction, and altered non-apoptotic cell-death programs. Ferroptosis and mitochondrial permeability transition (MPT)-driven necrosis may share upstream stress-related transcriptional signals, but their joint pathway-state features in cervical cancer remain incompletely characterized.

**Methods:**

We constructed a directionality-aware net pathway activity framework using evidence-audited ferroptosis-promoting, ferroptosis-inhibitory, MPT-promoting, and MPT-inhibitory gene sets in 304 TCGA-CESC primary tumors. The score was benchmarked against ssGSEA, GSVA, and PLAGE and evaluated in controlled simulations. For feature prioritization, score-defining genes were excluded from predictors and Elastic Net feature selection, XGBoost tuning, and model fitting were performed strictly within repeated nested cross-validation. Outer-test SHAP, model-sensitivity analysis, and a 200-permutation complete-pipeline test were used for interpretation and robustness assessment. External validation used GSE138080, GSE63514, GSE223804, and GSE151666.

**Results:**

CombinedNet showed moderate-to-high concordance with ssGSEA, GSVA, and PLAGE (Spearman rho = 0.735, 0.882, and 0.943, respectively), while 1,300 controlled simulations showed strong recovery of the latent promoter-minus-inhibitor balance across scenarios and effect sizes (mean rho = 0.944; mean sign-recovery accuracy = 0.902; mean standardized RMSE = 0.305). The leakage-resistant repeated nested-CV model yielded AUC = 0.780 (95% CI 0.766–0.795); a matched complete-pipeline permutation test gave empirical p = 0.005. Stability-weighted SHAP and perturbation prioritized *CKMT1B* and *KLK13*, whereas *GNA13*, *PANX2*, and all five sterol genes were not selected in any of 25 outer-fold models. The sterol genes retained modest score correlations but showed no model sensitivity. Across three lesion-progression cohorts, no pathway score remained associated with stage after FDR correction; in the cancer-only GSE151666 RNA-seq cohort, CombinedNet also showed no FIGO-stage trend.

**Conclusion:**

This study supports directionality-aware transcriptomic scoring and a leakage-resistant feature-prioritization framework, but does not support *GNA13*/*PANX2* or cholesterol-biosynthesis signals as stable model drivers or the composite score as a validated lesion-stage biomarker. The findings remain hypothesis-generating and require experimental validation.

## 1 Introduction

Cervical cancer remains a major global health burden and is primarily driven by persistent infection with high-risk human papillomavirus (HPV) (Sung et al., 2021; Wang et al., 2025; Sun et al., 2025; Tomaziu-Todosia et al., 2025; Inacio et al., 2023). HPV oncoproteins disrupt the p53 and retinoblastoma pathways and contribute to genomic instability, oxidative stress, and metabolic remodeling. These processes may reshape regulated cell-death programs during the transition from premalignant cervical lesions to invasive carcinoma.

Ferroptosis is an iron-dependent form of regulated cell death characterized by lipid peroxide accumulation and failure of antioxidant defense systems such as the *SLC7A11*-GSH-*GPX4* axis (Dixon and Olzmann, 2024; Liu et al., 2022; Koppula et al., 2021; Kang et al., 2021; Niu et al., 2024; Wang et al., 2022; Chen et al., 2024). Mitochondrial permeability transition (MPT)-driven necrosis is a mitochondrial stress-associated cell-death process involving opening of the mitochondrial permeability transition pore, collapse of mitochondrial homeostasis, and necrotic cell death (Bonora et al., 2022; Bernardi et al., 2022; Waseem and Wang, 2023; Llambi et al., 2016; Naim and Kaufmann, 2020; Neitemeier et al., 2017). Although these programs are mechanistically distinct, both can be influenced by oxidative stress, mitochondrial dysfunction, iron metabolism, and membrane lipid composition. Sterol metabolism, including 7-dehydrocholesterol and *DHCR7*-mediated cholesterol biosynthesis, has recently been linked to ferroptosis susceptibility and membrane lipid oxidation (Li et al., 2024; Freitas et al., 2024; Yamada et al., 2024; Lee et al., 2025). This broader non-apoptotic cell-death framework is consistent with current cell-death nomenclature and mitochondrial ferroptosis biology (Liu et al., 2023; Galluzzi et al., 2018).

In transcriptomic studies, pathway activity scoring and interpretable machine learning can be used to prioritize features associated with pathway-defined states (Miao et al., 2024; Ma et al., 2020). However, when labels are derived from transcriptomic pathway scores, model outputs should not be interpreted as independent clinical prediction. Instead, the model is best understood as a feature-prioritization framework for pathway-defined transcriptional states. In this study, we constructed a ferroptosis-MPT net activity framework, applied elastic net and XGBoost modeling to TCGA-CESC, interpreted the model using outer-test SHAP, evaluated model-sensitivity analyses, and assessed robustness across multiple independent GEO cohorts.

A central analytical issue in this setting is how to summarize pathway activity from bulk transcriptomic profiles without oversimplifying biologically bidirectional processes. Ferroptosis and MPT are not represented by uniformly pro-death genes alone; both pathways include genes that promote execution, genes that buffer oxidative or mitochondrial stress, and genes whose expression may reflect compensatory adaptation. A conventional enrichment score may therefore become difficult to interpret when promoting and inhibitory components are simultaneously upregulated. To address this problem, we used a directionality-adjusted net activity score, defined as the relative balance between promoting and inhibitory transcriptional components. This strategy does not measure biochemical pathway flux, but it provides an interpretable transcriptomic proxy for the balance between cell-death-promoting and cell-death-buffering states. Accordingly, the revised analysis benchmarks the directional formulation against established enrichment methods and controlled simulations rather than assuming that the z-score implementation is uniquely superior.

Interpretable machine learning was used because the relationship between cell-death pathway states and transcriptomic features is likely to be multivariable, nonlinear, and distributed across canonical and non-canonical genes. Penalized regression reduces dimensionality and stabilizes feature selection in highly correlated expression matrices, whereas gradient-boosting models can capture interactions that may not be apparent from univariate differential expression alone. SHAP analysis further allows model behavior to be decomposed into feature-level contributions, thereby converting a black-box classifier into a hypothesis-generating prioritization framework. In this study, the machine-learning model was not intended to serve as an independent clinical diagnostic test; rather, it was designed to identify transcriptional features associated with pathway-defined ferroptosis-MPT net activity states. Because the labels are mathematically derived from transcriptomic scores, model performance is treated as secondary and SHAP values are interpreted only as contributions to the pathway-defined binary state.

This framing is particularly suitable for a computational genomics audience because the main contribution lies in integrating public cancer transcriptomes, directionality-aware pathway scoring, interpretable machine learning, and external dataset validation. Rather than claiming experimental proof of a single causal mechanism, the study aims to prioritize genomic features and pathway modules that may guide future functional validation in HPV-associated cervical carcinogenesis.

## 2 Materials and methods

### 2.1 Data sources and preprocessing

TCGA-CESC RNA-seq data were obtained through the recount3/TCGA transcriptomic resource. The dataset initially contained cervical squamous cell carcinoma and endocervical adenocarcinoma samples, and the analysis focused on 304 primary tumor samples after exclusion of normal tissues and samples not meeting preprocessing criteria. Expression values were represented as log2(TPM+1).

GSE138080 (Agilent; Normal n = 10, HGCIN n = 15, Cancer n = 10), GSE63514 (Affymetrix; Normal n = 24, CIN1 n = 14, CIN2 n = 22, CIN3 n = 40, Cancer n = 28), and GSE223804 (RNA-seq; Normal n = 2, CIN n = 5, Cancer n = 9) were used to assess lesion-stage transportability. GSE151666 (RNA-seq; n = 68 pretreatment primary cervical cancers) was additionally used to assess score behavior across FIGO 2009 stages and candidate-gene context. Each cohort was processed and standardized independently within platform; RNA-seq and microarray expression matrices were not pooled or batch-corrected across cohorts because platform and biological-group effects were not separable. Gene coverage was documented for every cohort.

### 2.2 Ferroptosis-MPT net activity scoring

Ferroptosis- and MPT-related genes were re-audited for directionality using primary literature and public pathway knowledge. The evidence-audited primary score contained 32 genes: ferroptosis-promoting *ACSL4*, *LPCAT3*, *TFRC*, *TF*, *HMOX1*, *NOX1*, *CYBB*, *STEAP3*, *NCOA4*, *VDAC2*, *VDAC3*, *SLC1A5*, and *SLC38A1*; ferroptosis-inhibitory *SLC7A11*, *GPX4*, *FTH1*, *FTL*, *HSPB1*, *PRDX6*, *CISD2*, and *CISD1*; MPT-promoting *PPIF*, *BID*, *BAX*, *SLC25A4*, and *BOK*; and MPT-inhibitory *BCL2*, *BCL2L1*, *MCL1*, *BNIP3L*, *SIRT3*, and *HSP90AA1*. *DHCR7*, *MSMO1*, *HMGCR*, *SQLE*, and *FDPS* were analyzed separately as a sterol/cholesterol-biosynthesis module and were not used to define the primary score. The submitted 39-gene definition was retained as a sensitivity analysis.

For each sample, gene expression values were z-score standardized across samples within each cohort. FerroNet was defined as the mean z-score of ferroptosis-promoting genes minus the mean z-score of ferroptosis-inhibitory genes, and MPTNet was defined analogously. FerroNet and MPTNet were then standardized and CombinedNet was calculated as their mean. TCGA-CESC samples were stratified into High and Low activity groups using the median CombinedNet value.

The rationale for using a net activity score was to distinguish pathway activation from pathway compensation. For example, simultaneous elevation of lipid-peroxidation genes and antioxidant-defense genes may indicate an oxidative stress response rather than unopposed ferroptosis execution. Therefore, for each pathway, positive-direction genes and negative-direction genes were summarized separately before subtraction. The combined ferroptosis-MPT score was then calculated as the average of net ferroptosis and net MPT scores after scaling, so that samples with higher values represented a more death-prone transcriptomic state, whereas lower values represented a more buffered or resistant state. CombinedNet was therefore treated as an analytical composite of two distinct transcriptional axes, not as evidence of additive or synergistic biological interaction between ferroptosis and MPT.

Because pathway scores were derived from gene expression rather than direct metabolite, lipid-peroxide, mitochondrial membrane potential, or calcium measurements, they were interpreted as transcriptomic activity proxies. This distinction is important for computational genomics studies based on retrospective public data. The score was used to define reproducible transcriptional states within TCGA-CESC and to evaluate whether similar activity patterns could be observed in independent external cohorts.

To benchmark score construction, promoter and inhibitor gene sets were scored separately by ssGSEA, GSVA, and PLAGE; directional versions were calculated as promoter minus inhibitor, and PLAGE signs were oriented to promoter-gene expression. Concordance with the z-score net score was assessed by Spearman correlation and median-split High/Low agreement. In addition, 1,300 controlled simulations generated sample-level promoter and inhibitor latent effects under baseline, promoter-up, inhibitor-up, equal co-upregulation, and unequal co-upregulation scenarios. Recovery of the true directional balance (promoter minus inhibitor) was quantified by Spearman correlation, standardized RMSE, and sign-recovery accuracy; undirected union-gene-set enrichment was included as a sensitivity comparison.

### 2.3 Elastic net feature selection and XGBoost modeling

Elastic net logistic regression (alpha = 0.5) and XGBoost were implemented in a repeated stratified nested cross-validation framework (5 outer folds × 5 repeats). The 32 score-defining genes were excluded from the predictor matrix to reduce direct mathematical circularity. Within each outer-training set, variance filtering, scaling, Elastic Net feature selection, and XGBoost hyperparameter tuning were refitted using inner resampling; the untouched outer-test fold was used only for final prediction and interpretation. The primary Elastic Net analysis used lambda.min, and lambda.1se was evaluated as a prespecified sparsity sensitivity analysis. Model performance was summarized across outer folds by AUC, Brier score, and Cohen’s kappa and was treated as a secondary measure because the High/Low labels were derived from transcriptomic pathway scores.

Elastic net was chosen as the first modeling layer because tumor transcriptomes contain highly correlated gene-expression features, and pure univariate screening may select unstable markers driven by cohort-specific noise. The combined L1/L2 penalty allows correlated features to be retained in a controlled manner while shrinking weak and redundant predictors. A lambda.1se sensitivity analysis tested whether a more conservative feature set materially changed the conclusions.

The XGBoost model was then used as a nonlinear learner for activity-state discrimination. Hyperparameter tuning and model evaluation were performed within a nested cross-validation framework so that feature evaluation was separated from held-out performance estimation within the available sample size. Because the High/Low labels were derived from transcriptomic pathway scores, XGBoost and SHAP were used for nonlinear feature prioritization rather than as evidence of an independent diagnostic phenotype.

### 2.4 Outer-test SHAP analysis and model-sensitivity analysis

Outer-test SHAP values were calculated for each held-out fold using its corresponding outer-fold model and then aggregated across repeats. Candidate importance was summarized using mean absolute SHAP together with selection frequency, preventing single-fold high-SHAP features from being interpreted as stable priorities.

For model-sensitivity analysis, a candidate gene was perturbed only when it had been selected by the corresponding outer-fold model; its expression was replaced by the fifth percentile of the outer-training distribution and the change in predicted High-activity probability was recorded. Genes absent from a fold model were not artificially injected. Single-gene analyses included stable candidates and the five-gene sterol module (*DHCR7*, *MSMO1*, *HMGCR*, *SQLE*, and *FDPS*), with bootstrap confidence intervals.

Stable protein-coding candidates (selection frequency ≥0.60) were ranked using two prespecified axes: stability-weighted SHAP and absolute perturbation effect. Their mean percentile rank defined a consensus ranking. Biological relevance, Open Targets druggability/tractability annotations, and experimental accessibility were reported descriptively and did not influence rank.

### 2.5 Statistical analysis, permutation testing, and external validation

Continuous variables were compared using the Wilcoxon rank-sum test or Kruskal-Wallis test, as appropriate, and correlations were assessed using Spearman correlation. Multiple comparisons in score-stage and correlation families were controlled using the Benjamini-Hochberg false-discovery rate (FDR). A matched fixed 1 × 5 complete-pipeline permutation test used 200 label permutations and repeated variance filtering, scaling, Elastic Net selection, XGBoost tuning, refitting, and prediction under shuffled labels; empirical p values used (1 + number of null AUCs ≥ observed AUC)/(1 + number of permutations). External cohorts were analyzed independently using the locked score formula; Kruskal-Wallis, Jonckheere-Terpstra trend tests, Dunn *post hoc* tests with BH correction, and effect sizes with confidence intervals were reported. Two-sided p < 0.05 was considered nominally significant for exploratory analyses.

## 3 Results

### 3.1 Directionality-aware scoring was robust to alternative methods and simulation

Using the evidence-audited 32-gene definition, FerroNet and MPTNet represented distinct transcriptional axes (Spearman rho = −0.357, p = 1.5 × 10^-10). For CombinedNet, correlations with directional ssGSEA, GSVA, and PLAGE were 0.735, 0.882, and 0.943, with median-split High/Low concordance of 78.3%, 88.2%, and 92.1%, respectively (Figure 1A). The primary 32-gene and submitted 39-gene definitions also showed concordant CombinedNet values in TCGA (rho = 0.756; High/Low concordance = 80.3%), and the significance status of external-cohort comparisons did not change under the submitted definition.

**FIGURE 1:**
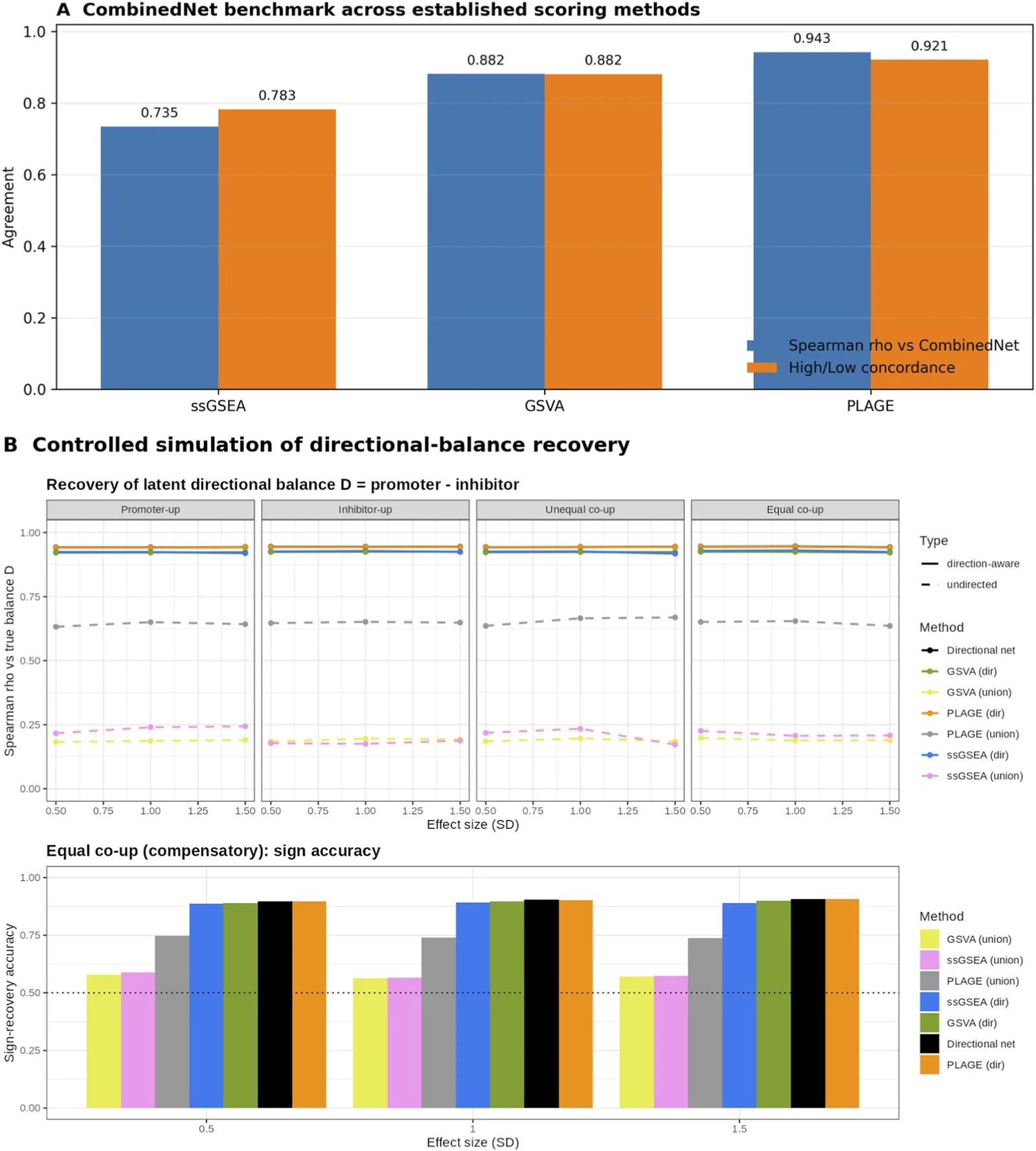
Score-method benchmark and controlled simulation. **(A)** CombinedNet benchmark against directional ssGSEA, GSVA, and PLAGE, showing Spearman correlation with the z-score CombinedNet and median-split High/Low concordance. **(B)** Controlled simulations comparing direction-aware and undirected union-set methods for recovery of the latent promoter-minus-inhibitor balance, including equal and unequal co-upregulation scenarios.

Across 1,300 simulations spanning baseline and four perturbation scenarios, the directional net score recovered the latent promoter-minus-inhibitor balance with mean Spearman rho = 0.944, mean sign-recovery accuracy = 0.902, and mean standardized RMSE = 0.305 across scenario/effect-size combinations. Directional ssGSEA, GSVA, and PLAGE performed similarly, whereas undirected union-set versions showed substantially poorer recovery, including when promoter and inhibitor genes were co-upregulated (Figure 1B). These results support the signed promoter-minus-inhibitor formulation but do not indicate that the specific z-score engine is uniquely superior to other direction-aware scoring methods.

### 3.2 Leakage-resistant nested resampling identified a stable feature core

Feature selection was repeated within every inner training loop rather than once on the full cohort. Across 25 outer-fold fits, *LIPM*, CMB9-22P13.1, *CKMT1B*, *KLK13*, and *TRPC1* were selected in 25/25 folds, with *ESRRA* and *CKMT1A* selected in 24/25 folds. *CKMT1B* and *KLK13* combined complete selection stability with the largest stability-weighted SHAP values among reproducible features (Figures 2A,B). The primary lambda.min analysis selected a median of 145 features per fold; the lambda.1se sensitivity analysis produced a much sparser median of 44 features per fold while preserving similar AUC (0.775 versus 0.780; Figure 2C).

**FIGURE 2:**
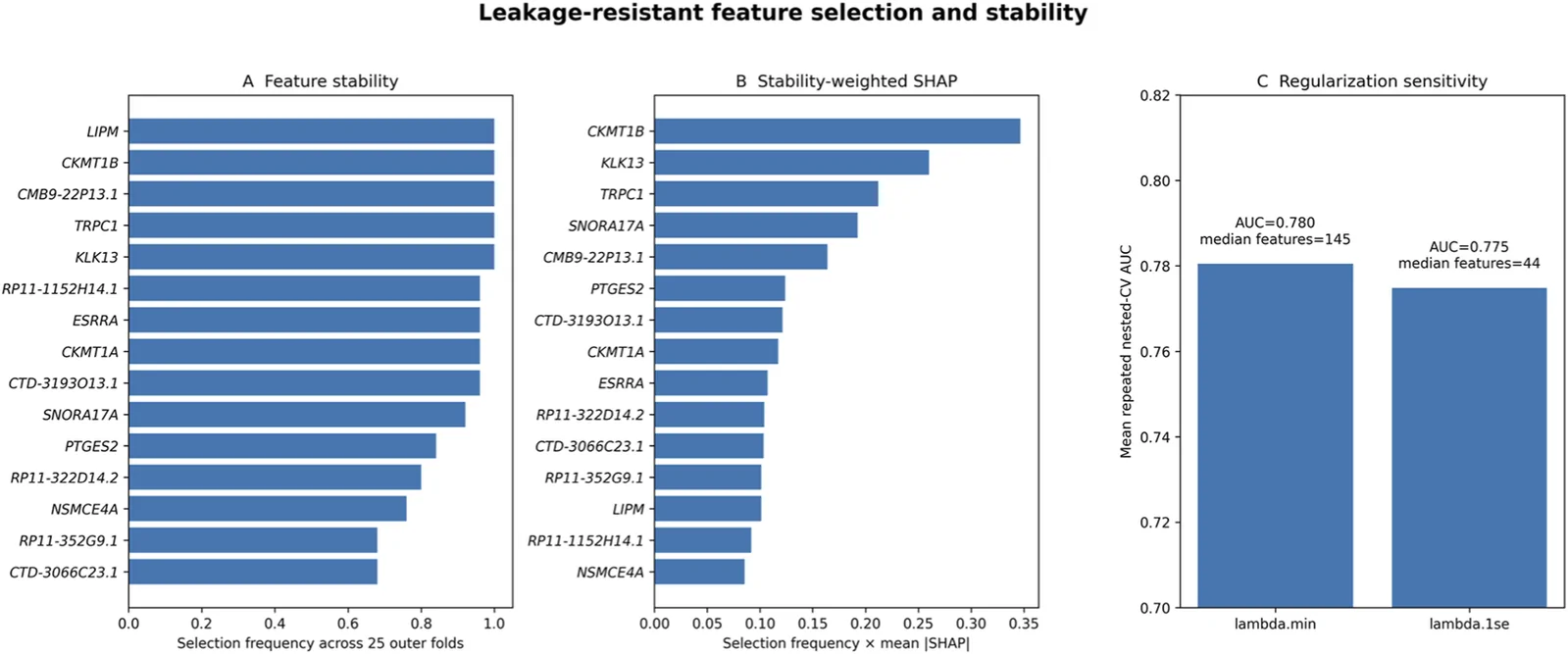
Leakage-resistant feature selection and stability. **(A)** Selection frequency across 25 outer-fold fits for the most stable features. **(B)** Stability-weighted SHAP ranking. **(C)** Sensitivity of repeated nested-CV AUC and model sparsity to lambda.min versus lambda.1se regularization.

### 3.3 Classifier performance exceeded chance but was treated as secondary

The repeated 5 × 5 nested-CV model yielded mean AUC = 0.780 (95% CI 0.766–0.795), Brier score = 0.207 (95% CI 0.198–0.216), and Cohen’s kappa = 0.426 (95% CI 0.372–0.481; Figures 3A,B). In the matched fixed 1 × 5 design used for permutation testing, observed AUC was 0.771. None of 200 complete-pipeline label permutations reached this value (null mean = 0.494; null maximum = 0.600), yielding empirical p = 0.005 (Figure 3C). These values indicate non-random discrimination of the transcriptome-derived activity labels, but they are not presented as clinical predictive performance.

**FIGURE 3:**
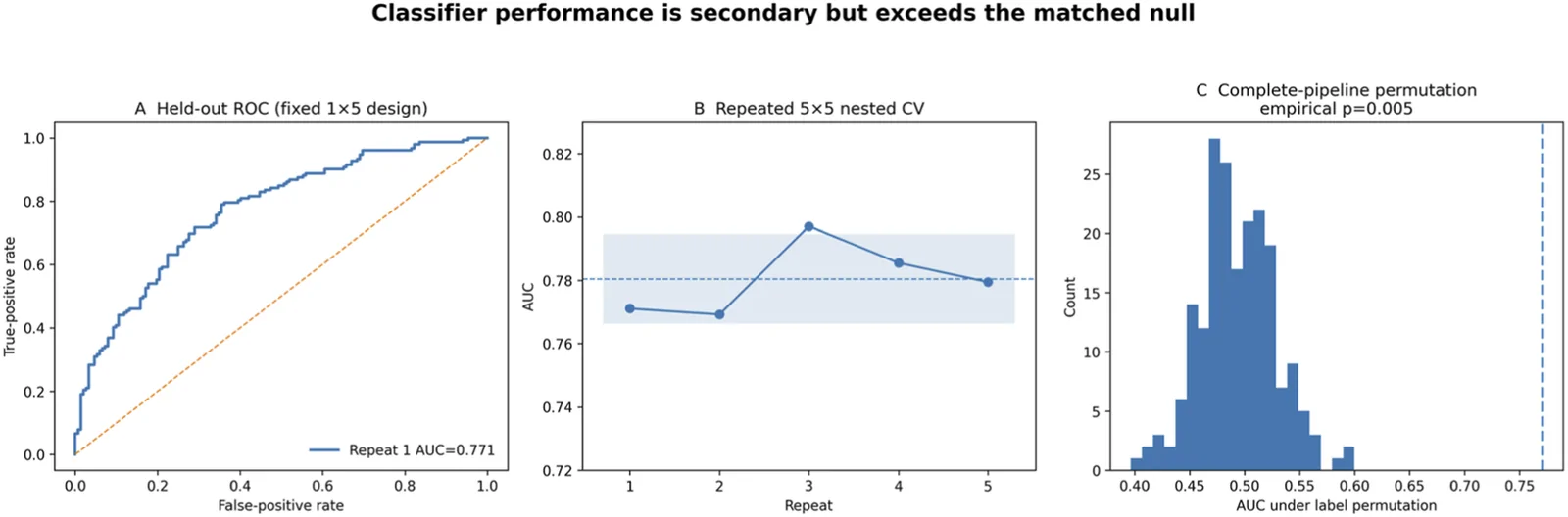
Classifier performance and complete-pipeline permutation test. **(A)** Held-out ROC curve for the fixed 1 × 5 design used for permutation testing. **(B)** AUC across five repeats of nested 5-fold cross-validation; the dashed line and shaded band indicate the mean and 95% confidence interval. **(C)** Null AUC distribution from 200 complete-pipeline label permutations; the dashed vertical line indicates the observed AUC. Performance is interpreted as secondary evidence for feature prioritization rather than clinical prediction.

### 3.4 Stability-weighted SHAP and model-sensitivity prioritized *CKMT1B* and *KLK13*

Among 22 stable protein-coding candidates, *CKMT1B* and *KLK13* were Tier A candidates based on the combined stability-weighted SHAP and perturbation ranking (Figure 4; Table 1). *NSMCE4A*, *ESRRA*, *PTGES2*, and *TRPC1* followed in the consensus ranking and had varying degrees of tractability annotation. These ranks are intended to prioritize follow-up experiments rather than establish causal pathway regulators.

**FIGURE 4:**
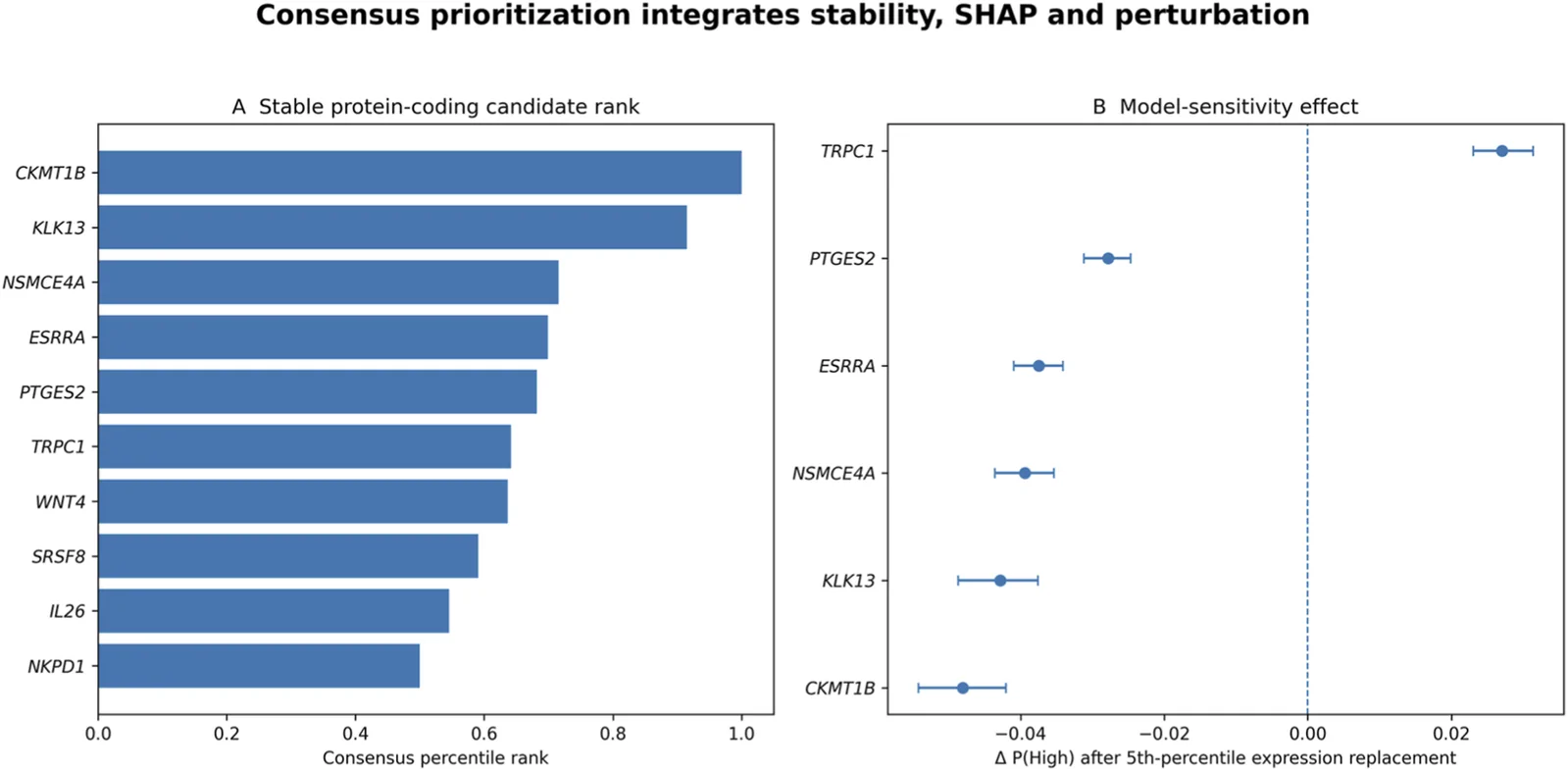
Consensus candidate prioritization. **(A)** Consensus percentile ranking of stable protein-coding candidates based on stability-weighted SHAP and perturbation-effect ranks. **(B)** Model-sensitivity effects with bootstrap confidence intervals for candidates with estimable perturbation effects. Druggability and tractability annotations are reported in Table 1 and do not influence the rank.

**TABLE 1:** Top ranked stable protein-coding candidates integrating stability-weighted SHAP and model-sensitivity results.

Ranked candidate	Selection frequency	Mean |SHAP|	Perturbation ΔP(High)	Tier	Biological/experimental context	Druggability/tractability
1. CKMT1B	1.00 (25/25)	0.347	−0.048	A	Mitochondrial creatine kinase; energetic coupling	Not assessed
2. KLK13	1.00 (25/25)	0.260	−0.043	A	Secreted serine protease; extracellular assay	No small-molecule; secreted protein
3. NSMCE4A	0.76 (19/25)	0.112	−0.039	C	SMC5/6 complex; genome stability	No annotation
4. ESRRA	0.96 (24/25)	0.112	−0.037	B	Metabolic nuclear receptor; mitochondrial programs	Ligand/structure; druggable family
5. PTGES2	0.84 (21/25)	0.147	−0.028	B	PGE synthase; lipid/redox signaling	High-quality ligand
6. TRPC1	1.00 (25/25)	0.212	+0.027	B	Cation channel; Ca2+ signaling	Ligand/structure; druggable family

Reviewer-nominated *GNA13* and *PANX2* were specifically re-examined. Neither gene was selected in any of 25 outer-fold models. In TCGA, *GNA13* showed moderate co-expression with several score genes, including *GCLC* (rho = 0.424), whereas *PANX2* showed weaker-to-moderate correlations, including with *GCLC* (rho = 0.308). Neither gene was associated with overall survival, and the queried STRING network did not identify direct edges connecting either gene to the score-gene network. *GNA13* varied across lesion stages in GSE63514, but this did not reproduce in the small GSE223804 cohort. The previous HPV-stratified SHAP interpretation was removed from the main analysis because the HPV-negative subgroup could not support reliable inference; the originally submitted visualization is retained only as Supplementary Figure S3 for transparency.

### 3.5 Model-sensitivity analysis did not support a cholesterol-biosynthesis driver

Model-sensitivity effects for stable candidates were small: *CKMT1B* and *KLK13* showed mean changes in predicted High-activity probability of −0.048 and −0.043, respectively, and all candidate effects were approximately |Δ| ≤ 0.05. By contrast, *DHCR7*, *MSMO1*, *HMGCR*, *SQLE*, and *FDPS* were selected in 0/25 outer-fold models; under the prespecified provenance rule they were therefore not injected into models and had no estimable perturbation effect (Figure 5B).

**FIGURE 5:**
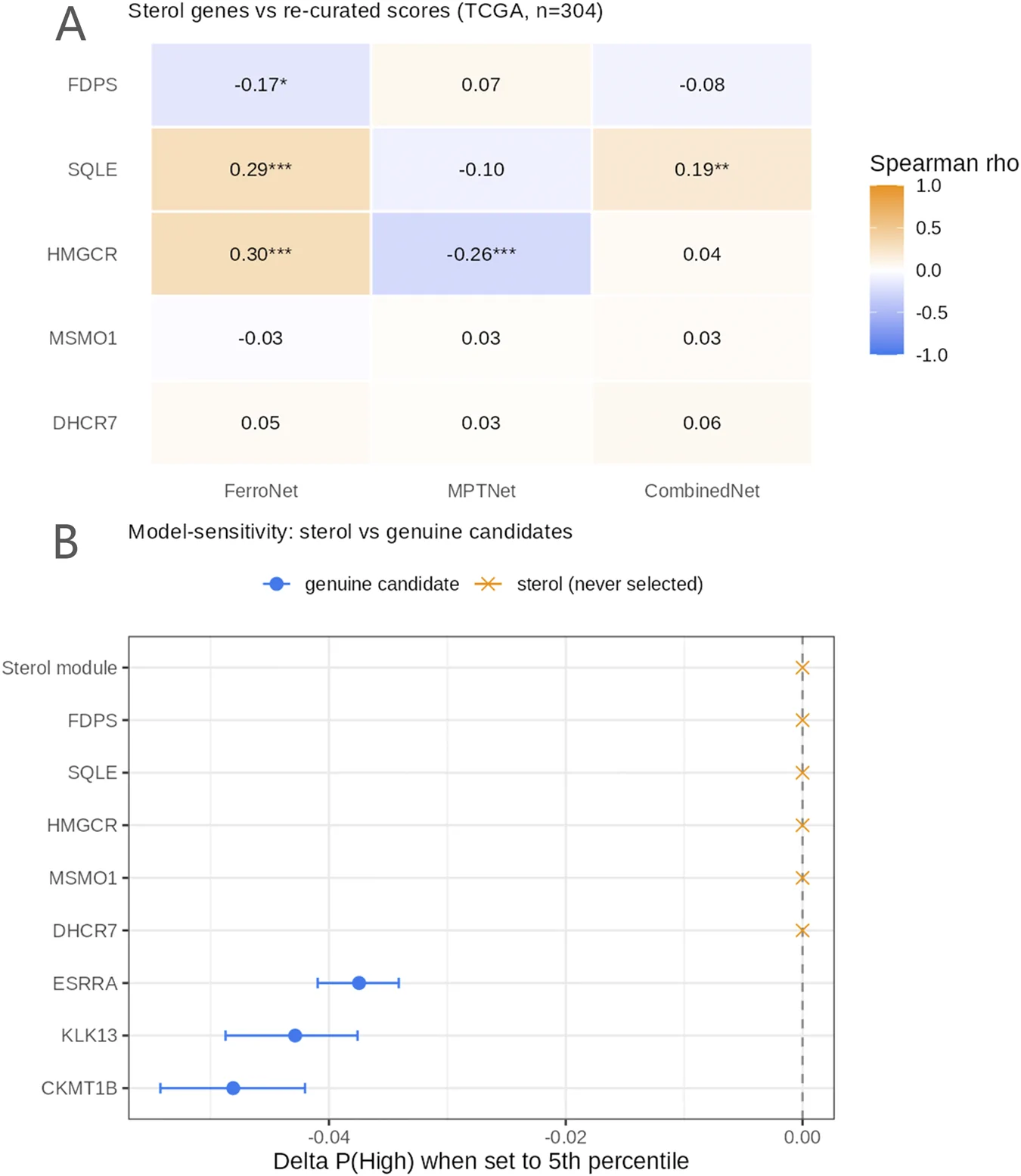
Sterol/cholesterol-biosynthesis context. **(A)** Spearman correlations of *DHCR7*, *MSMO1*, *HMGCR*, *SQLE*, and *FDPS* with FerroNet, MPTNet, and CombinedNet; asterisks indicate BH-adjusted significance. **(B)** Model-sensitivity comparison showing that the five sterol genes and the sterol module were not selected in any outer-fold model, whereas representative stable candidates had small but estimable effects.

The sterol genes nevertheless showed transcriptomic correlations with pathway scores. *HMGCR* correlated with FerroNet (rho = 0.299) and MPTNet (rho = −0.259), *SQLE* with FerroNet (rho = 0.294) and CombinedNet (rho = 0.188), and *FDPS* with FerroNet (rho = −0.166), with the reported associations surviving BH correction where indicated in Figure 5A. Thus, sterol metabolism remains an exploratory biological context, but the revised analysis does not support the five-gene module as a stable model driver or functional dependency.

### 3.6 External validation did not support lesion-stage stratification

Using the locked scoring definition and within-cohort standardization, no pathway score showed a significant stage effect after BH correction in GSE138080, GSE63514, or GSE223804 (minimum omnibus FDR = 0.669; Figure 6). No pairwise stage contrast remained significant after correction, and effect-size confidence intervals were broad, particularly in GSE223804 (Normal n = 2). Therefore, the originally observed HGCIN ferroptosis pattern was not reproduced as a robust cross-cohort trajectory.

**FIGURE 6:**
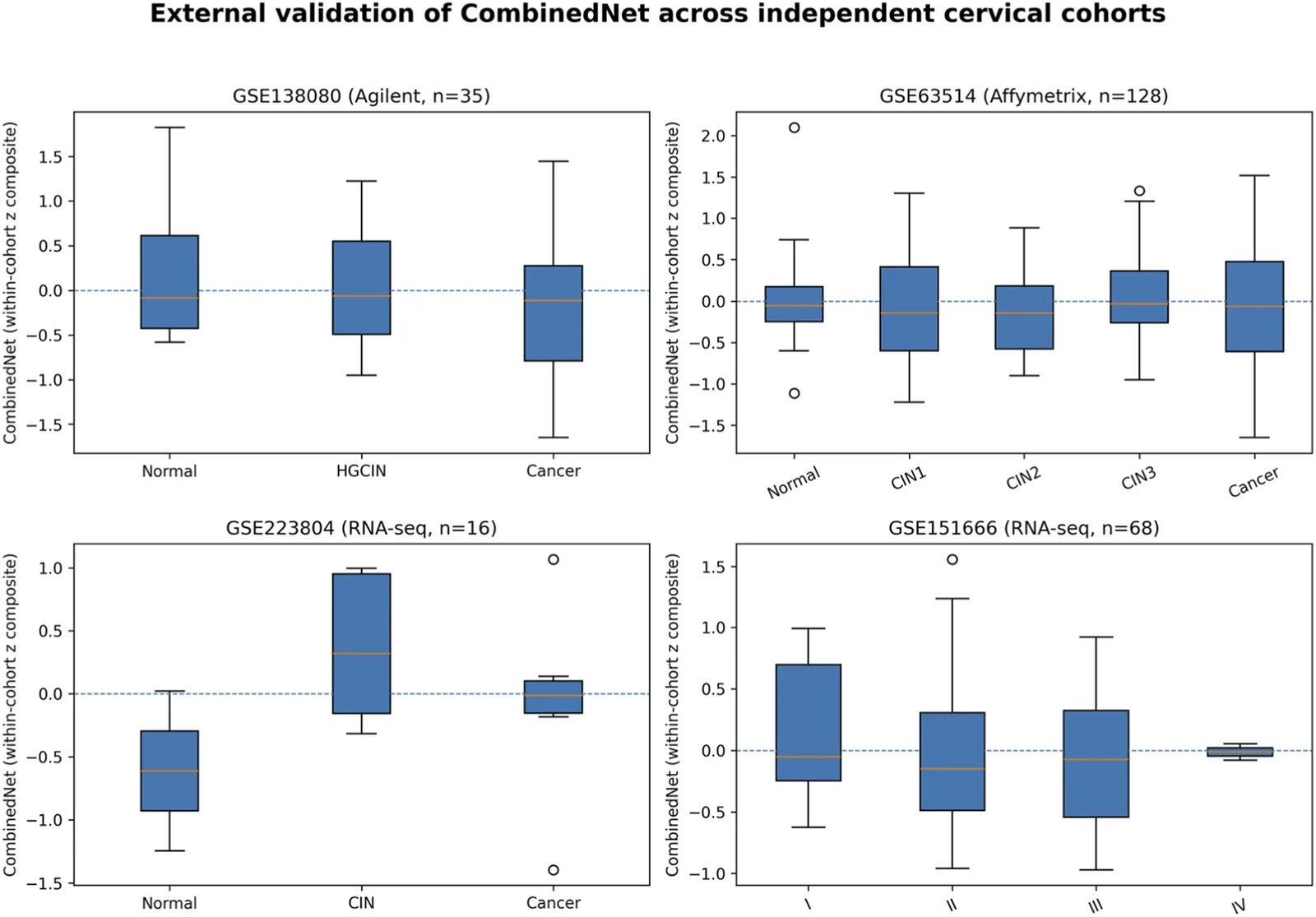
External validation of CombinedNet across independent cervical cohorts. CombinedNet distributions are shown for GSE138080, GSE63514, GSE223804, and GSE151666 using within-cohort standardization. No lesion-stage comparison remained significant after BH correction in the first three cohorts, and GSE151666 showed no significant FIGO-stage trend. Full FerroNet, MPTNet, effect-size, and pairwise statistics are provided in the Supplementary Material.

GSE151666 provided an additional independent RNA-seq assessment in 68 pretreatment cervical cancers with complete 32/32 score-gene coverage. CombinedNet did not vary significantly across FIGO stages (Kruskal-Wallis p = 0.674; Jonckheere-Terpstra p = 0.476). Exploratory candidate-gene analyses showed positive correlations of *CKMT1B* (rho = 0.433) and *CKMT1A* (rho = 0.438) with CombinedNet, providing limited independent support for candidate-score association but not for lesion-stage prediction.

## 4 Discussion

This study reframes the analysis as an interpretable computational genomics investigation of ferroptosis-MPT net activity states in cervical cancer. Instead of claiming experimental mechanism, the revised framework prioritizes transcriptomic features associated with pathway-defined activity labels. This positioning is better aligned with the Computational Genomics section of Frontiers in Genetics and reduces the risk of over-interpreting machine-learning results as functional validation.

From a computational genomics perspective, the value of this work lies in converting public transcriptomic data into biologically interpretable activity-state models. The study integrates directionality-aware pathway scoring, benchmark and simulation analyses, leakage-resistant nested machine-learning evaluation, outer-test SHAP interpretation, model-sensitivity analysis, permutation testing, and independent GEO validation. This combination matches the aims of computational genomics research: extracting structured biological information from large-scale genomic datasets, developing reproducible analytical frameworks, and generating testable hypotheses from public resources.

The negative correlation between FerroNet and MPTNet (rho = −0.357) reinforces that the two components should not be interpreted as uniformly co-activated. CombinedNet is an analytical composite of two separately standardized transcriptional proxies, not an additive or synergistic biological interaction term. Ferroptosis and MPT remain mechanistically distinct cell-death axes that may share upstream stress biology but can vary independently across tumors.

The benchmark and simulation analyses also refine the rationale for the net score. Directional ssGSEA, GSVA, and PLAGE showed moderate-to-high concordance with the z-score implementation, and all direction-aware methods recovered the simulated promoter-minus-inhibitor balance well. In contrast, undirected union-set enrichment lost directional information when promoting and inhibitory genes were co-upregulated. Accordingly, the methodological contribution is the explicit signed construction rather than a claim that mean z-score subtraction is intrinsically superior to established enrichment algorithms.

Within the revised leakage-resistant pipeline, *CKMT1B* and *KLK13* emerged as the most consistent Tier A candidates because they combined high selection stability, high stability-weighted SHAP, and measurable but modest model-sensitivity effects. Other leading stable protein-coding candidates included *NSMCE4A*, *ESRRA*, *PTGES2*, and TRPC1. *CKMT1A*, although lower in the consensus ranking, was selected in 24/25 folds and showed an independent positive correlation with CombinedNet in GSE151666. These observations remain prioritization signals rather than mechanistic evidence.

Importantly, the previously emphasized *GNA13* and *PANX2* signals were not reproduced as stable ML features after score re-auditing and leakage-resistant resampling. Although *GNA13* retained moderate co-expression with several score genes and showed a lesion-stage association in GSE63514, neither *GNA13* nor *PANX2* was prognostic, neither was selected in any outer-fold model, and no direct STRING connection to the queried score-gene network was identified. We therefore removed mechanistic language and the underpowered HPV-stratified SHAP interpretation from the main analysis. These genes are retained only as exploratory co-expression observations requested by the reviewer, while the original HPV-stratified visualization is retained solely as Supplementary Figure S3 without mechanistic inference.

The sterol/cholesterol-biosynthesis analysis likewise changed substantially. *HMGCR*, *SQLE*, and *FDPS* showed modest correlations with one or more transcriptomic activity scores, which is biologically compatible with literature linking sterol intermediates and ferroptosis susceptibility (Li et al., 2024; Freitas et al., 2024; Yamada et al., 2024). However, all five prespecified sterol genes were absent from every outer-fold model, so the revised analysis provides no model-sensitivity evidence for the sterol module. This distinction between correlation and model dependence is important: the sterol signal should be considered a preliminary contextual hypothesis, not a validated regulatory module or therapeutic dependency.

External validation further limits the scope of inference. After applying the locked score independently in GSE138080, GSE63514, and GSE223804 with BH correction, no consistent stage-associated pattern was detected. GSE151666 similarly showed no CombinedNet trend across FIGO stages. These negative results indicate that the score may describe heterogeneity among TCGA tumors but is not currently supported as a progression or staging biomarker. Cross-platform differences and small lesion strata remain relevant sources of uncertainty, but the lack of replication is retained as a substantive finding rather than interpreted as confirmation.

Several limitations should be acknowledged. First, High/Low labels are mathematically derived from transcriptomic pathway scores, so the ML model does not represent independent clinical prediction; excluding score-defining genes and using nested resampling reduce but do not eliminate this conceptual circularity. Second, bulk transcriptomic data cannot measure biochemical ferroptosis or mitochondrial permeability transition and cannot resolve cell-type-specific activity. Third, *in silico* expression replacement is a model-sensitivity analysis and does not reproduce genetic knockdown or pharmacological inhibition. Fourth, the candidate ranking depends on one discovery cohort and remains exploratory. Fifth, external cohorts differ in platform, tissue processing, disease spectrum, and sample size, and GSE223804 includes only two normal samples.

Finally, HPV-stratified inference is especially limited. The originally used HPV-negative subgroup was extremely small and could not be robustly reconstructed from the revised TCGA provenance; accordingly, HPV subgroup SHAP claims were removed from the main analysis. For transparency and to follow the reviewer’s request, the originally submitted HPV-stratified SHAP visualization is retained only as Supplementary Figure S3 and is not used for mechanistic or subgroup-specific inference. GSE151666 HPV comparisons are retained only descriptively. Prospective functional studies in well-characterized HPV-positive and HPV-negative cervical models will be required to determine whether the prioritized candidates have pathway-specific biological effects.

## 5 Conclusion

This study provides an interpretable computational genomics framework for directionality-aware characterization of ferroptosis-MPT transcriptomic states and for leakage-resistant feature prioritization in cervical cancer. Benchmarking and simulation support the signed promoter-minus-inhibitor formulation, while strict nested resampling and permutation testing identify *CKMT1B* and *KLK13* as stable candidate features. Conversely, the revised analysis does not support *GNA13*/*PANX2* or the five-gene sterol module as stable model drivers, and independent cohorts do not validate the score as a lesion-stage biomarker. The resulting candidate list and pathway-state framework should therefore be viewed as hypothesis-generating resources for future functional validation.

## Data Availability

The original contributions presented in the study are included in the article/Supplementary Material, further inquiries can be directed to the corresponding author.

## Supplementary material

SUPPLEMENTARY FIGURE S1 Revised analytical workflow. Public transcriptomic datasets, directionality audit and scoring, score benchmark and controlled simulation, leakage-resistant nested machine learning, permutation/model-sensitivity analyses, and external validation are shown in sequence.

SUPPLEMENTARY FIGURE S2 Reviewer-requested *GNA13*/*PANX2* lesion-stage context. Expression patterns across GSE63514 and GSE223804 are shown as exploratory context; neither gene was a stable feature in the revised nested-CV models, and no mechanistic inference is drawn.

SUPPLEMENTARY FIGURE S3 Exploratory HPV-stratified SHAP visualization from the originally submitted analysis. The HPV-negative subgroup contained only nine samples; therefore, this panel is retained solely for transparency as exploratory supplementary material and is not used for mechanistic, subgroup-specific, or confirmatory inference.
